# Exosomes as smart drug delivery vehicles for cancer immunotherapy

**DOI:** 10.3389/fimmu.2022.1093607

**Published:** 2023-01-17

**Authors:** Huan Zhang, Simiao Wang, Man Sun, Yaxin Cui, Jianming Xing, Lesheng Teng, Zhifang Xi, Zhaogang Yang

**Affiliations:** ^1^ School of Life Sciences, Jilin University, Changchun, China; ^2^ School of Horticulture and Food, Guangdong Eco-Engineering Polytechnic, Guangzhou, China

**Keywords:** exosomes, immunotherapy, drug delivery system, immune checkpoint blockade, tumor immune microenvironment

## Abstract

Exosomes (Exos) as drug delivery vehicles have been widely used for cancer immunotherapy owing to their good biocompatibility, low toxicity, and low immunogenicity. Some Exos-based cancer immunotherapy strategies such as tuning of immunosuppressive tumor microenvironment, immune checkpoint blockades, and cancer vaccines have also been investigated in recent years, which all showed excellent therapeutic effects for malignant tumor. Furthermore, some Exos-based drug delivery systems (DDSs) for cancer immunotherapy have also undergone clinic trails, indicating that Exos are a promising drug delivery carrier. In this review, in order to promote the development of Exos-based DDSs in cancer immunotherapy, the biogenesis and composition of Exos, and Exos as drug delivery vehicles for cancer immunotherapy are summarized. Meanwhile, their clinical translation and challenges are also discussed. We hope this review will provide a good guidance for Exos as drug delivery vehicles for cancer immunotherapy.

## 1 Introduction

Cancer has become a major cause of death worldwide. According to the latest statistiscs, there will be a total of more than 1.9 million new cancer patients and 600 thousand cancer deaths in the United States in 2022, suggesting that cancer has seriously threatened human health (1, 2). Although traditional therapeutics, including radiation, chemotherapy and surgery, have shown a certain tumoricidal ability, there are still some limitations (3, 4). These therapeutics often kill both cancer and normal cells, leading to severe side effects and drugs resistance (5). Therefore, it is critical to find an effective therapeutic approach with low or no side effects (6).

**Figure 1:**
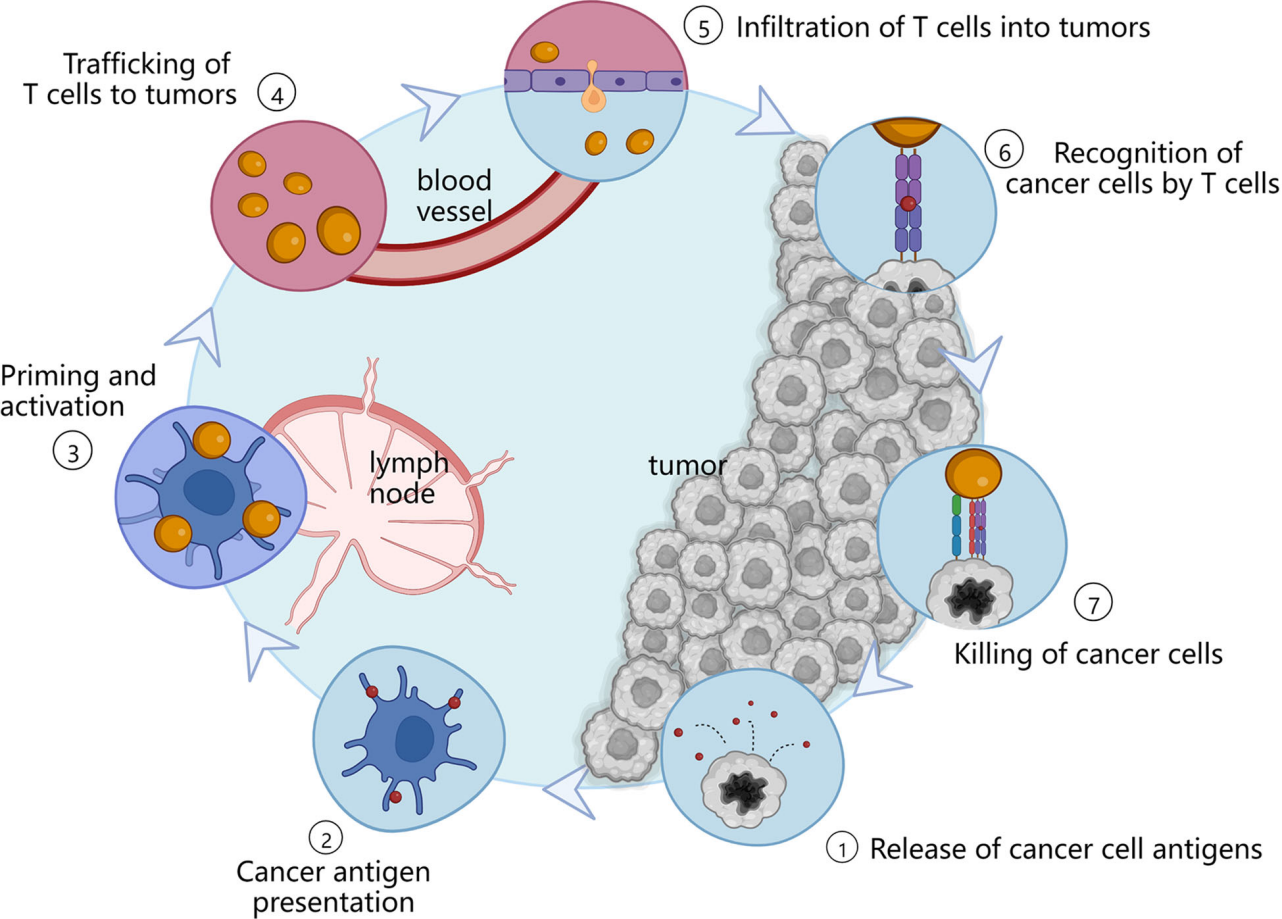
Immune actions in the cancer immunity cycle. APCs present antigens which are released by tumor cells to lymphocytes. And then, these antigens can be subsequently recognized by T cells located in the lymph nodes, thereby activating specific immune responses to the tumor sites.

Cancer immunotherapy is a novel therapeutic approach that exploits the body’s own immune system to recognize and eradicate tumor cells (3, 7). In order to achieve sustained antitumor immune response, the cancer immunity cycle must be repeatedly initiated and expanded (3), as shown in **Figure 1**. Firstly, tumor cells release some tumor-specific immunogenic antigens, and then, antigen-presenting cells (APCs) including dendritic cells (DCs) and macrophages present antigens for the activation of certain lymphocytes *via* major histocompatibility complex I (MHC-I). After that, these antigens can be further recognized by T cells including CD4^+^ T cells and CD8^+^ T cells inside the lymph nodes, and thus resulting in specific immune responses to the cancer cells. In this case, cancer immunotherapy can specifically kill cancer cells with minimal effect to normal cells, and induce immunological memory to trigger long-term protection against tumor recurrence (5, 8). Therefore, cancer immunotherapy has attracted widespread attentions in the field of cancer therapy.

Nowadays, a series of cancer immunotherapy approaches including nonspecific immune stimulation (9), immune checkpoint blockades (ICB) (10), and cancer vaccines (11, 12) have been evaluated to modulate immune responses. Moreover, some cancer immunotherapy drugs including cytotoxic T-lymphocyte-associated protein 4 (CTLA-4) inhibitors (10), programmed cell death 1 (PD-1) inhibitors and programmed cell death 1 ligand 1 (PD-L1) inhibitors have been authorized by the United States Food and Drug Administration (FDA) for clinical use (13, 14). Although these inhibitors have shown exciting outcomes, some shortcomings still exist. For instance, many malignant tumors have the ability of releasing different immunosuppressive molecules into the tumor microenvironment (TME), promoting their immune escape or suppressing immune reactions (15). Furthermore, their therapeutic effect is often diminished by off-targeting delivery, the induction of immune tolerance and evasion, and all these limit their applications (13, 16). In order to overcome these shortcomings, many researchers focus on the application of drug delivery systems (DDSs). DDSs can deliver payloads including immune checkpoint inhibitors (ICIs) and immunosuppressive regulatory molecules to the desired site and realize the sustained release of the drugs, thereby improving the efficiency of cancer immunotherapy. Currently, various DDSs, such as exosomes (Exos), liposomes, and nanoparticles, have been extensively studied and hold great promise in cancer immunotherapy.

Exos, one of drug delivery carriers, are 40–160 nm sized extracellular vesicles secreted by live cells and can be found in different types of biological fluids (e.g., serum, saliva, and urine) (17). They possess many advantages such as small size, good biocompatibility, low toxicity, and low immunogenicity (18). Meanwhile, Exos can protect cancer immunotherapeutic agents from degradation, thus increasing their circulation time and targeting ability (19). Unlike liposomes and other synthetic drug nanoparticle carriers, Exos are able to inherit the properties of parent cells and obtain some components of parent cells such as proteins, lipids and nucleic acids, which may endow them homing effect and the ability to activate immune responses (20). Moreover, Exos contain transmembrane and membrane anchored proteins, which may enhance target cells’ endocytosis and promote the delivery of their internal content (16). In addition, Exos could be easily engineered to improve drug-loading capacity and tissue-specific targeting (21). Therefore, Exos are recognized as a promising drug carrier.

In order to promote the development of Exos in cancer immunotherapy, in this review, we comprehensively summarized the application of Exos as smart drug delivery vehicles for cancer immunotherapy. First, the biogenesis and composition of Exos are introduced. Then, Exos as drug carrier for cancer immunotherapy are discussed. Finally, the clinical translation and challenges of Exos as drug delivery vehicles are presented.

## 2 Exosomes

**Figure 2:**
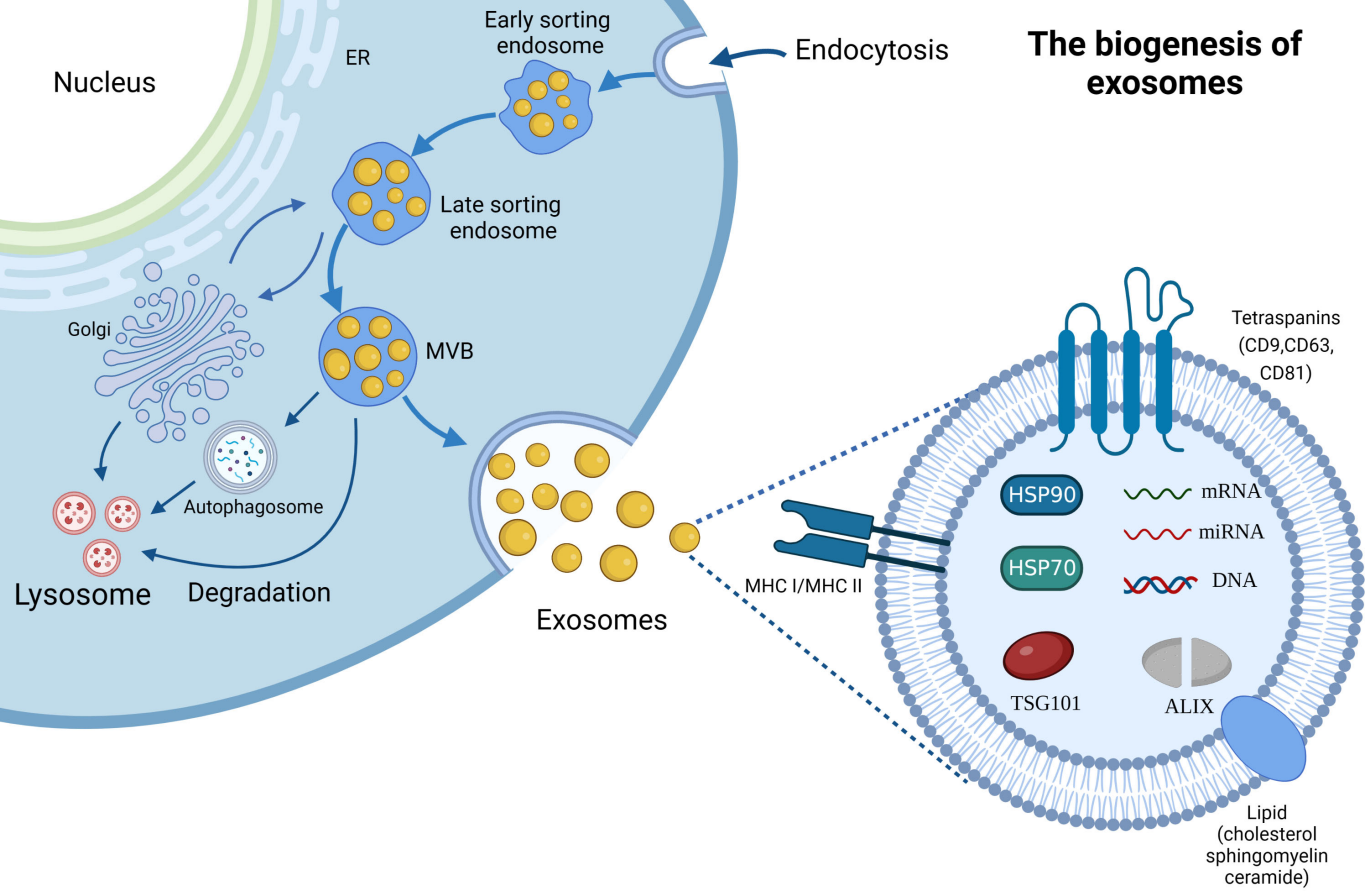
The biogenesis and composition of Exos. ER; Endoplasmic reticulum, MVB; Multivesicular Body.

The name “exosome” (Exo) first appeared in 1981. At that time, Trams et al. (22) extracted plasma membrane-derived vesicles with 5’-nucleotidase activity, and referred the vesicles as Exos. Exos are the important subset of extracellular vesicles, possessing 40–160 nm particle size (23). A large number of researches have proven that Exos can be actively secreted by most, if not all, organisms including bacteria (24) and almost all cell types (e.g., red blood cells (25), platelets (26), immune cells (27), fibroblasts (28), endothelial cells (29), epithelial cells (30) and tumor cells (31). Their secretion mechanism is simple, and the scheme is shown in **Figure 2**. It is generally recognized that the generation of Exos involves three major steps: invagination, multivesicular bodies generation, and secretion (32, 33). The generation of Exos begins with the inward budding of the plasma membrane and generates several endocytic vesicles which encapsulate proteins both on the surface of the plasma membrane and in the extracellular matrix (32). And then, early sorting endosomes (ESEs) are formed under the effect of the endocytic sorting complex and the proteins required for transport. After that, ESEs mature into late sorting endosomes, and continue inward invagination to form multivesicular bodies (MVBs). Finally, MVBs, which contain many intraluminal vesicles (ILVs), can either fuse with the cytoplasmic membrane to release Exos into the extracellular environment or fuse with lysosomes or autophagosomes to be degraded.

It is generally believed that the biogenesis of Exos is a tightly controlled process. In brief, two potential mechanisms are involved in this process: endosomal sorting complexes required for transport (ESCRT) dependent mechanism and ESCRT-independent mechanism (34). Of which, ESCRT provides a crucial mechanism for the formation and sorting of the ILVs (35). ESCRT consists of a five-part protein complex with different roles including ESCRT-0, -I, -II, -III and the AAA ATPase Vps4. Specifically, ubiquitin-binding ESCRT-0 binds directly to specific structural domains of the endosomal membrane through the action of hepatocyte growth factor-regulated tyrosine kinase substrate (HRS) with endosomal-specific phosphatidylinositol 3-phosphate (PtdIns-3-P) (36). Then, ESCRT-I and ESCRT-II are recruited by the interaction between HRS and the ESCRT-I subunit TSG101 (37), and the complexes further recruit ESCRT-III which consists of various soluble coiled-coil-containing proteins Vps2, Vps20, Vps24, and Snf7 to form a protein complex which is involved in promoting the inward budding processes (38). The ESCRT-III complex drives vesicle division and is dissociated and recovered from the MVB membrane through the mediation of the AAA ATPase Vps4 (39). An increasing number of studies demonstrated that several ESCRT-related proteins can affect the secretion of Exos (40, 41). For example, the experimental results of twenty-three components of the ESCRT machinery in Exos biogenesis and related proteins in MHC II-expressing HeLa cells by RNA interference (RNAi) have shown that silencing of HRS, STAM1 and TSG101 can reduce secretion of Exos and decrease the expression of MHC II and CD63 proteins (40, 41). Meanwhile, silencing of VPS4B increased secretion of exosome marker proteins (CD63, MHC II, HSC70), and depletion of ALIX enhanced MHC II-expression on Exos and secreting cells (40). Another research also showed that ESCRT-III-associated protein ALIX interacts with cytoplasmic adaptor syntenin, thus promoting the intraluminal budding of endosomal membrane and Exos’ secretion (42). Likewise, the depletion of ESCRT-III and its associated proteins, including CHMP4C, VTA1, increased Exos’ secretion (42).

Exos are regarded as small “progeny” of parental cells, because it contains components of parental cells. They contain thousands of proteins, lipids and nucleic acids, and the scheme is presented in **Figure 2**. Typically, Exos contain a variety of non-specific proteins, including heat shock proteins (HSP70, HSP90), membrane transport proteins (such as annexins and flotillin), cytoskeletal proteins (myosin, actin and tubulin), MHC proteins (MHC I and MHC II) (43), adhesion molecules (CD11b and CD54) (44), and tetraspanins protein superfamily (CD9, CD63 and CD81) which is considered as the marker protein of Exos (45). Moreover, ALIX and TSG101 proteins aforementioned are also the important components of Exos. Cell type-specific proteins have also been discovered in Exos, such as the A33 protein secreted by the human colon tumor cell line LIM1215 (46), further suggesting that their composition is related to the type and physiological condition of the source cells. In addition, Exos also possess extensive lipids, cholesterol, sphingomyelin, glycosphingolipids and different patterns of RNAs including mRNAs and non-coding RNAs (e.g., miRNAs, circRNAs, lncRNAs, ribosomal RNAs (rRNAs) and transfer RNAs (tRNAs)) (47, 48). Of which, bioactive lipids play an important role in the stability and structural rigidity of Exos, cholesterol can regulate Exos’ secretion, and sphingomyelin triggers calcium influx (47). Meanwhile, exosomal miRNAs, such as miR-214, miR-29a, miR-1, miR-126, and miR-320, participate in angiogenesis, hematopoiesis, exocytosis, and tumorigenesis (48). Moreover, exosomal lncRNAs as an intercellular signaling are also involved in the development of oncogenesis and regulation of the TME.

## 3 Exos as drug delivery carrier for cancer immunotherapy

### 3.1 The source of Exos

Exos, especially these secreted from tumor cells and immune cells, may influence the phenotype and immune function of target cells (49). In order to better understand the source of Exos as drug delivery carrier, in this section, we summarized the characteristics of immune cell-derived and tumor cell-derived Exos (TEXs).

#### 3.1.1 Immune cells-derived Exos

Immune cells mainly include DCs, macrophage, B lymphocytes, T lymphocyte cells, etc. Phagocytes (e.g., macrophages and neutrophils) and natural killer (NK) cells act as the first line of defense against pathogens, rapidly activating the innate immune response and killing pathogens; T cells, B cells and related cytokines can activate specialized humoral and cellular immune responses, respectively (50). However, Exos produced by immune cells are able to inherit the properties of parent cells and participate in the innate and adaptive immune responses (20). Therefore, a large number of researches have used immune cells-derived Exos as drug carriers for cancer immunotherapy.

DCs are classical APCs that stimulate specific antigenic immune responses (23). DCs-derived Exos (DEXs), which mainly contain MHC-I, MHC-II, costimulatory molecules (CD80 and CD86), heat shock proteins (HSP70 and HSP90) and adhesion molecules (ICAM-1) (51), are the most widely used immune cells-derived drug carrier. They can activate T cells to kill cancer cells through directly binding of MHC-peptide complex and costimulatory molecules to T cell receptors (TCR) (51). Moreover, DEXs also can present the MHC-peptide complex to another DCs which is possibly an inactivated DC, thereby increasing the expression of the MHC-peptide complex, and subsequently leading to large-scale activation of T cells (52). In fact, DEXs have the same therapeutic effect as the parent DCs. For example, genetically modified DEXs contain Th2 cytokines (e.g., IL-4 and IL-10) and apoptotic proteins (e.g., FASL) to inhibit inflammation and ameliorate the extent of collagen-induced arthritis (53). In contrast, NK cells derived Exos contain NK markers like CD56, NKG2D, CD94, CD40L and killer proteins (e.g., FASL and perforin) (54). NK cells-derived Exos can induce tumor cells apoptosis by significant activation of caspase death pathways *via* perforin and FASL (55, 56). In addition to killer proteins, NK cell-derived Exos may also carry tumor suppressor miRNAs such as miR-186, and thus inhibiting tumor growth and TGFβ1-dependent immune escape, and all of which exhibited the therapeutic potential of NK cell-derived Exos (57).

Macrophage-derived Exos, another immune cells-derived Exos, exhibit pro-inflammatory and pro-tumor functions, which mainly depend on the phenotype of macrophages (M1 and M2 subtypes) (58, 59). For instance, M1 phenotype macrophages-derived Exos (M1-Exos) can activate NLRP3 inflammasomes to enhance the cytotoxicity of T cells and NK cells and thus inhibiting the growth of tumor (20). Moreover, they can also upregulate the expression of miRNAs (e.g., miR-146a, miR-146b and miR-21-3p) and resolve inflammation by inhibiting NF-κB and TLR signaling pathways (60, 61). Meanwhile, a study has proven that M1-Exos can repolarize M2 tumor associated macrophages (TAMs) to M1 macrophages, resulting in pro-inflammatory cytokines releasing and synergistic effects of anti-PD-L1 in tumor immunotherapy (62). In contrast, M2 phenotype macrophages derived Exos showed the ability to suppress T-cell function and participate in tumor proliferation, migration, angiogenesis, and facilitate tumor immune escape (63).

In addition, B lymphocytes and T lymphocyte cells are also immune cells used for Exos generation. B lymphocytes derived−Exos contain CD19, B cell-specific markers, and the immunogenic molecules (e.g., MHC-I, MHC-II, CD40, CD54 and CD86), which stimulate T lymphocytes proliferation, activation and T(H)2-like cytokine production (64–66). Meanwhile, T cell-derived Exos express TCR, adhesion factors and various markers including CD2, CD3, CD4, CD8, CD11c, CD25, CD69, LFA-1, CXCR4, FASL, GITR (67). In general, T lymphocyte cells are classified into two phenotypes: CD4^+^ T cells and CD8^+^ T cells (68). Depending on their functions and the expression of antigens, CD4^+^ T cells are further classified as regulatory T cells (Tregs), Th cells and follicular helper T cells (Tfhs) (67). The Exos secreted by different phenotypes T cells have distinct regulatory effects on immune cells and non-immune cells (67). For example, Exos purified from CD8^+^ T cells generate proliferation in autologous resting cells and produce a higher proportion of CD8^+^ T cells (69). CD8^+^ cytotoxic T lymphocyte (CTL)-derived Exos have a potent benefit when used as DDSs for tumor immunotherapy since the inherited CTL properties. Exos derived from IL-12-stimulated CTLs could directly activate naive CD8^+^ T cells in the absence of antigen, producing IFN-γ and granzyme B, and eliminating tumor cells (70, 71). Conversely, Treg-derived Exos contain specific molecular cargo (let-7b, let-7d, miRNA-155 and iNOS) and cooperate with cytokines (IL-10 and TGF-β) to perform immunosuppressive functions (72).

Furthermore, other immune cell-derived Exos as drug delivery carrier, including neutrophil-derived Exos (73), mast cell-derived Exos (74), eosinophils-derived Exos (75) and myeloid-derived suppressor cell-derived Exos (76), also showed an essential role in the immune microenvironment, participating in immune regulation, inflammatory responses, intercellular communication, etc. (77, 78).

#### 3.1.2 Tumor-derived Exos

In general, TEXs are rarely used as drug carriers for cancer immunotherapy, which mainly because they accurately reproduce the content of parent tumor cells (79–82), and transfer oncogenic signals including activated oncoproteins, transcripts, oncogenic DNA sequences and oncogenic micro-RNAs (83–85) to surrounding immune cells, stromal cells and other tumor cells, and induce various functional changes in the cells (86–89). However, TEXs also contain some immunostimulatory molecules, such as CD80, CD86, MHC complexes (90, 91). They can act as adjuvants and participate in antigen presentation, and thus stimulating the activation of immune response (92). For example, TEXs serve as effective carriers of the chemotherapeutic drug methotrexate and simultaneously act as immunomodulators, stimulating the recruitment of large quantity of neutrophils to the cholangiocarcinoma tumor region and activating the neutrophil anti-tumor response to alleviate obstructive extrahepatic cholangiocarcinoma (93). TEXs are also important mediators in intercellular communication and immune regulation, and the ability of TEXs to protect internal proteins or nucleic acids from degradation makes TEXs the most promising choice as diagnostic and prognostic biomarkers (94). Currently, TEXs are widely used as diagnostic biomarkers for non-small cell lung cancer (95), pancreatic cancer (96), colorectal cancer (97), and gastric cancer (98).

### 3.2 Drug-loading strategy

Various studies have suggested that exosome is a potential drug delivery carrier due to its high biocompatibility, low toxicity, low immunogenicity and the ability of crossing natural barriers (99). Various drug-loading strategies have been designed and developed, including incubation (100), physical loading techniques (e.g., electroporation, ultrasound and extrusion) (101, 102), and cell engineering techniques (103), etc. Their pros and cons are presented in **Table 1**.

**Table 1:** The pros and cons of exosomes-based drug-loading strategies.

Drug-loading strategies	Pros	Cons	Ref
Incubation	Simple operation; No special equipment required; Preservation of exosome integrity; Little damage to exosomes and drugs	Low drug loading efficiency; Cause cytotoxicity	(99, 100, 104, 105)
Ultrasound	High drug loading efficiency	Exosome membrane damage	(106–109)
Extrusion	High drug loading efficiency; Uniform exosome particle size	After reintegrating exosome integrity damage	(110–113)
Electroporation	High drug loading efficiency	Exosome aggregation; Require process optimization; Damage to exosome integrity	(101, 102, 107, 114)
Cell Engineering Techniques	Well-established operating strategy; Toxicity reduction	Complicated operation; Uncertainty of exosomal contents and the amount of cargo	(103, 115–117)

Incubation is the simplest drug-loading method, where the drug diffuses into the exosome membrane or cell membrane according to a concentration gradient (99). Up to now, three incubation strategies have been developed: direct incubation, transfection reagent-mediated incubation, and source cell-mediated incubation (118). They present multiple advantages such as simple operation, no special equipment requirement, preservation of exosome integrity and with minimum damage to Exos and drugs, and these drug-loading strategies have been applied to load different types of drugs. For example, study showed that Exo^Ce6+R848^ was constructed by simple co-incubation of HEK 293T cell-derived Exos with Chlorin e6 (Ce6) and immune adjuvant R848 to reprogram immunosuppressive M2-like phenotypic macrophages and restore the immune microenvironment (119).

Although some payloads cannot be loaded by co-incubation, commercial reagents with better transfection efficacy (e.g., lipofectamine and dharmaFECT3) have been applied to load drugs into Exos (120, 121). For instance, PD-L1 siRNA can be entrapped by Lipofectamine^®^ 2000, and then, adding Exos inside can decrease its cytotoxicity and improve its targeting (120). In addition, source cell-mediated incubation was also utilized to obtain DDSs (122). Specifically, donor cells were co-incubated with drugs, causing the secretion of Exos loaded with active drug components (123). Although incubation presents many advantages, the variety of encapsulated drugs is limited and the drug-loading efficiency is relatively low (124).

**Figure 3:**
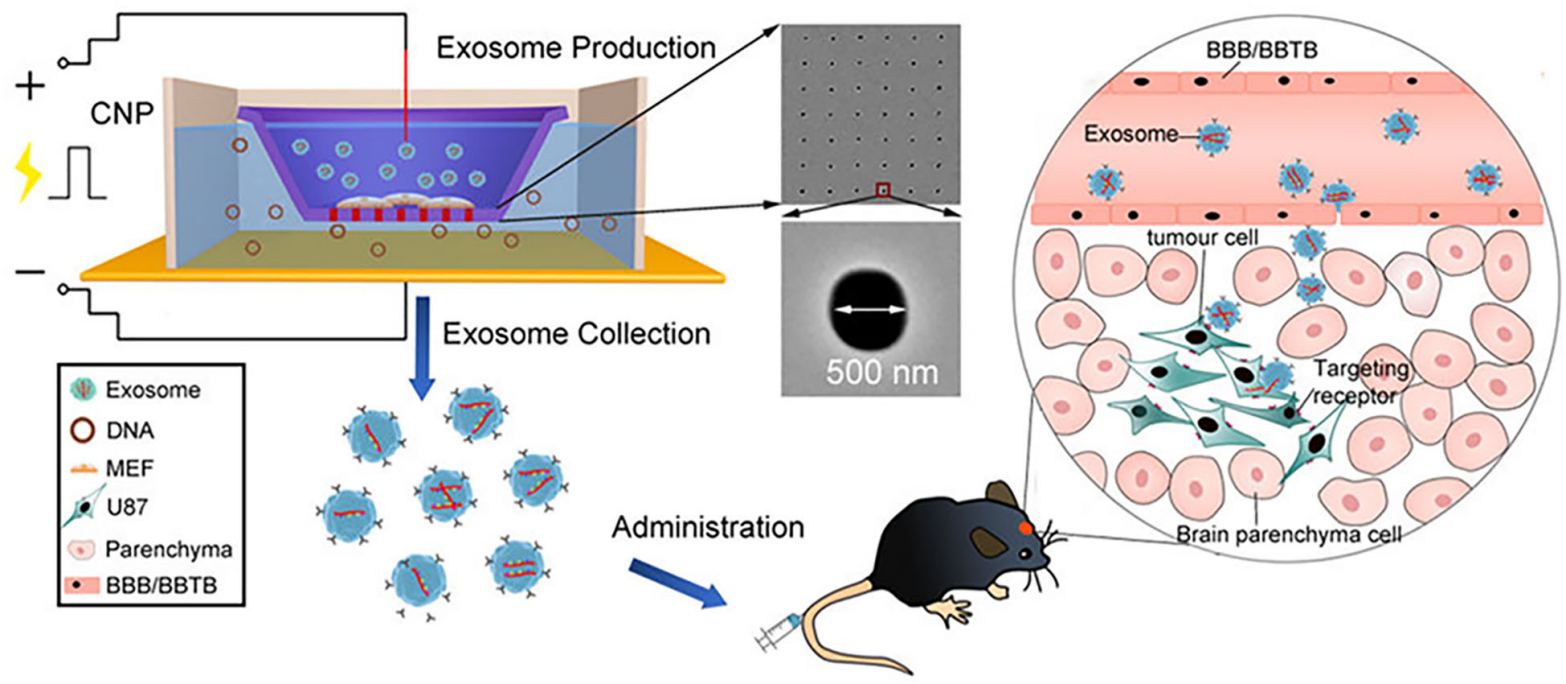
Schematic representation of CNP-generated EVs for targeted nucleic acid delivery (Reprinted with the permission from Ref. 131). Abbreviation: CNP: Cellular Nanoporation; BBB: Blood Brain Barrier; BBTB: Blood Brain Tumor Barrier.

Physical loading methods including electroporation, ultrasound and extrusion have also been widely applied to load drugs (125). Electroporation is a strategy that drugs are instantaneously loaded into Exos under an electrical impulse (126). In this situation, when the transmembrane potential reaches a certain threshold, a hydrophilic channel is formed in the membrane allowing small molecules hydrophilic nucleic acids to be rapidly loaded by the electric field, followed by self-healing of membranes, which can improve the drug-loading efficiency (127). In view of this, bone marrow mesenchymal stem cell (BM-MSC) Exos were loaded with galectin-9 siRNA by electroporation and modified with oxaliplatin (OXA) prodrug as an immunogenic cell death trigger to disrupt tumor immunosuppression by M2 TAMs and recruit cytotoxic T lymphocytes, achieving significant therapeutic efficacy in the immunotherapy of pancreatic ductal adenocarcinoma (128). In another study, it was indicated that the loading efficiency of electroporation was three times higher than that of normal incubation (129). Although electroporation showed high drug-loading efficiency, it may damage the intact structure of the Exos and cause cargo aggregation. Therefore, in order to solve these shortcomings, various innovative electroporation strategies were developed to load cargo into Exos (130, 131). Chang et al. (130) batch-produced a 3D NEP chip with a uniform and parallel nanochannel array. The results indicated that this chip showed a significant higher efficiency and transfection uniformity. In addition, our groups (131) developed a cellular nanoporation (CNP) biochip with 500 nm nanochannels, and the scheme is presented in **Figure 3**. In this work, pores were produced in the cell membrane under transient electrical pulses, and DNA plasmids were shuttled from the buffer into cells. The experimental results indicated that this approach causes less cellular damage and produces more than 50-fold Exos than that of conventional strategies. Moreover, more than 1000-fold mRNA transcripts were loaded inside compared to control. Different from this, the ultrasound method allows the drug to enter the Exos *via* disrupting the Exos membrane by mechanical shear (132). However, ultrasound can result in a degree of membrane damage (106). Extrusion is a technology that breaks the exosome membrane by external force, allowing the mixture of Exos and drugs to recombine into a new exosome (110). Though physical loading methods have been widely used, they also exist some limitations, such as damage the stability and integrity of Exos, specialized equipment requirement and limitation of production scale (118).

In addition to above-mentioned approaches, cell engineering technology is also a drug-loading method. It is a technology that modify the donor cells through gene editing technology or other methods to secrete Exos with target proteins on the cell surface. This approach is the most well-established and complex method, and has been extensively applied to load cargo into Exos (133, 134). Yong et al. (117) developed Exosome-sheathed doxorubicin-loaded PSiNPs (DOX@E-PSiNPs) generated by exocytosis of the tumor cells after treatment with DOX-loaded porous silicon nanoparticles (PSiNPs), penetrating deep into the tumor and exhibiting significant tumor toxicity. In spite of the wide range of applications and greater scope for manipulation of cellular engineering modifications, there are still limitations, such as complicated operations and uncertainty about the cargo of Exos and the amount of cargo (135).

### 3.3 Exos-based cancer immunotherapy strategies

Exos as a promising drug carrier show the advantage of good biocompatibility, low toxicity, and low immunogenicity. Exos-based cancer immunotherapy strategies including tuning of immunosuppressive tumor microenvironment (ITME), ICB, and cancer vaccines have been widely applied, as shown in **Table 2**. In order to better understand these strategies, their research status was summarized below.

**Table 2:** The samples of Exos-based DDSs for cancer immunotherapy.

DDSs	Exos source	Disease	Kind of study	Immunotherapy strategy	Immunotherapy efficacy	Ref
PTX-M1-Exos	M1-polarized macrophages	Breast cancer	*In vivo;* breast xenograft tumors model	Tuning of ITME	High anti-tumor effects	(109)
Exo@DOX–EPT1	Milk	Oral squamous cell carcinomas	*In vivo;* oral squamous cell carcinoma xenograft tumors model	pH targeting and tuning of ITME	High effectively treat oral squamous cell carcinomas	(136)
cGAMP@dual-antiExos	Melanoma cell	Melanoma	*In vitro*; B16F10 cells	ICB	Effectively activating immune response and inhibiting of immune escape	(137)
Exos encapsulated with sonosensitizers Ce6 and immune adjuvant R848	HEK293T cells	Prostate cancer	*In vivo;* mouse brain inflammatory model	Tuning of ITME	Activating effector T cells and reverting the immunosuppressive microenvironment	(119)
Engineered multifunctional immune-modulating Exos	Expi293F cell	Triple negative breast cancer	*In vivo*, NOD.Cg-Prkdcscid Il2rgtm1Wjl/SzJ (NSG) mice model	ICB	Activating T cells and eliciting robust anticancer immunity, and thus killing cancer cells	(138)
CpG-SAV-exo	Tumor cell	Murine melanoma	*In vivo*, B16BL6 tumor-bearing mice model	Antigen presentation and T-cell activation	Presenting stronger *in vivo* antitumor effects in B16BL6 tumor-bearing mice	(9)
Exos loaded CD62L and OX40L	HEK293T cells	Metastatic breast cancer	*In vitro*; 4T1 syngeneic mouse model	Tuning of ITME	Activating effector T cells and inhibiting Treg induction, and inhibiting tumor development	(139)
iEXO-OXA	Bone marrow mesenchymal stem cell	Pancreatic cancer	*In vivo*, Rthotopic PANC-02/luci tumor-bearing mice model	Tuning of ITME	Achieving significant therapeutic efficacy in cancer treatment	(128)
Exos with MART-1 peptide and CCL22 siRNA	Immunogenically dying tumor cells	Bladder cancer	*In vivo*; bladder cancer mice model	Cancer vaccines	High anti-tumor effects	(140)
Exos	CAR-T cell	Triple-negative breast cancer	*In vivo*; triple-negative breast cancer model	T-cell activation	Showing a highly effective tumor inhibition rate	(141)
SMART-Exos	Expi293 cells	Breast Cancer	*In vitro*; breast cancer cells (HCC 1954 cells)	T-cell activation	Showing a highly effective tumor inhibition rate	(142)

PTX-M1-Exos; M1-exosomes loading paclitaxel, ITME; Immunosuppressive tumor microenvironment, Exo@DOX–EPT1; Exosome-doxorubicin-anthracene endoperoxide derivative, ICB; Immune checkpoint blockades, Ce6; Chlorin e6, CpG-SAV-exo; CpG-SAV-exosomes, iEXO-OXA; Exosomes losding oxaliplatin; Exos, Exosomes.

#### 3.3.1 Tuning of immunosuppressive tumor microenvironment

As is known to us, TME is very complex and comprised of multiple components including cytokines, inflammatory cytokines, extracellular matrix and blood vessels, etc. (3). It plays an important role in the recruitment of immune cells and tumor progression (143). However, some cancer cells may evade immune systems due to the downregulation of tumor associated antigens, high infiltration of multiple immunosuppressive cells such as TAMs, and low expression of antitumor cytokines (144). In addition, both the physicochemical properties of cancer cells (e.g., hypoxia and weak acidity) and the abnormal metabolic activities can also promote the immune escape of tumors, resulting in an ITME, which becomes one of the major obstacles in cancer immunotherapy (136). Therefore, tuning of ITME can efficiently enhance cancer immunotherapeutic effects.

**Figure 4:**
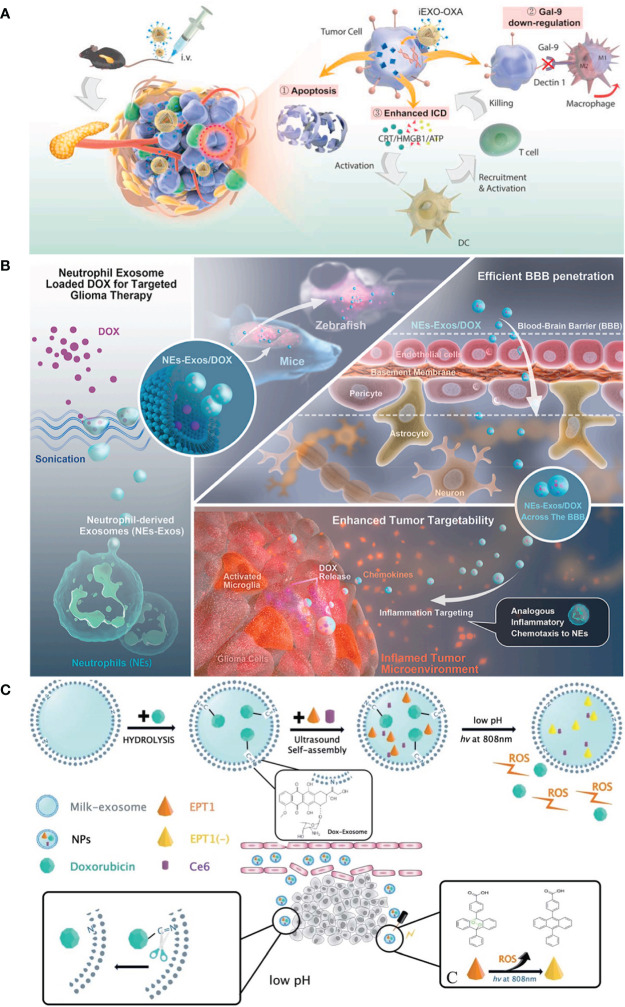
**(A)** Pancreatic-targeting exosomes-based dual delivery biosystem for pancreatic immunotherapy and reprogramming tumor microenvironment (Reprinted with the permission from Ref. 128); **(B)** The Scheme of NEs-Exos system for glioma immunotherapy (Reprinted with the permission from Ref. 147); **(C)** The preparation scheme and therapeutic process of Exo@DOX–EPT1 for oral squamous cell carcinoma (Reprinted with the permission from Ref. 136). iEXO-OXA, Exosomes losding oxaliplatin; ICD, immunogenic cell death; EPT1, Endoperoxide derivative; NPs, Nanoparticles; DOX, Doxorubicin; ROS, reactive oxygen; Ce6, Chlorin e6.

TAMs, essential elements of the immune responses in TME, play a critical role in inhibiting tumor growth and metastasis (6). TAMs were divided into two phenotypes: tumor-suppressing M1 macrophages and tumor-promoting M2 macrophages. In general, TME promotes the functionality of TAMs into M2 phenotypes, and M2 macrophages produce immunosuppressive cytokines to facilitate tumor progression (145). In contrast, M1 macrophages are activated by pro-inflammatory cytokines, resulting in tumor suppression (146). Thus, regulation of macrophage polarization from M2 phenotypes to M1 phenotypes can efficiently inhibit cancer progression. In order to reactivate TME and enhance the efficiency of breast cancer therapy, Zhao et al. (6) designed and established exosome delivery system derived from M1 macrophage (DTX-M1-Exo). The results indicated that DTX-M1-Exo can promote the development of naïve macrophages into M1 phenotype. Meanwhile, M1 macrophages was long-term maintained by modulating mitochondrial function. DTX-M1-Exo showed a great antitumor therapeutic efficacy. Similarly, Zhou et al. (128) designed and developed a pancreatic-targeting Exos-based dual delivery biosystem (iEXO-OXA) for pancreatic immunotherapy, and the scheme is shown in **Figure 4A**). In their work, Exos were secreted from bone marrow mesenchymal stem cell. Galectin-9 siRNA was loaded inside Exos by electroporation method, and OXA was modified on the surface to trigger immunogenic cell death. The results indicated that iEXO-OXA promoted the polarization of M2 phenotype to M1 phenotype upon disrupting the combination of galectin-9 and dectin 1, and TME was reprogrammed, increasing anti-tumor immunity for pancreatic cancer. Moreover, researches showed that high molecular-weight folic acid could suppresses M1 macrophage polarization and enhance M2 polarization, resulting in immunosuppression (15). In order to modulate TME, Feng et al. (15) designed and fabricated folic acid modified exos with expressing of human hyaluronidase (PH20) drug delivery platform (Exos-PH20-FA) for cancer therapy. The results indicated that Exos-PH20-FA can degrade high molecular-weight folic acid to low-molecular-weight folic acid and polarize macrophages to the M1 type, thereby improving the efficiency of cancer therapy. Meanwhile, Exos-PH20-FA also reduced tumor cell metastasis, which provides a promising treatment for cancer.

Persistent inflammation is also another characteristic of TME (146). It can induce stromal destruction and normal tumor vasculature, and thus inhibiting tumor growth. Researches showed that the secretion of pro-inflammatory cytokines, such as TNF-α, can trigger the apoptosis of cancer cells in tumor site (148). Wang et al. (109) established DDSs based on M1-EXOs. The results indicated that the expression level of caspase-3 and pro-inflammatory cytokines were elevated when M1-Exos were exposed around macrophages. Macrophages were polarized to M1 phenotype, and thus enhancing antitumor activity. In addition, in recent years, inflammatory TME targeting has been recognized as a promising and attractive therapeutic strategy. Encouraged by these, Wang et al. (147) designed and developed a neutrophil-Exos (NEs-Exos) system to deliver DOX for glioma immunotherapy, and the scheme is shown in **Figure 4B**). First, they isolated Exos from neutrophil by ultracentrifugation technique. And then, DOX was loaded inside Exos by sonication. The cellular uptake and the effect of NEs-Exos *in vitro* were investigated. In addition, the tumor-targetability and anti-glioma effect of NEs-Exos were also examined *in vivo*. The results indicated that NEs-Exos not only present the ability of crossing blood brain barrier, but can also respond to inflammatory stimuli and move to inflamed glioma site.

In addition, owing to high glycolysis rate and increased production of lactate, weak acidity becomes another distinct hallmark of ITME, and it can induce irreversible tumor metastasis and promote the tumor growth (149). Therefore, many researchers were devoted to develop pH-responsive DDSs to target tumors and improve tumor therapy efficiency. Kim et al. (149) fabricated a pH-responsive DDSs based on i-motif-modified Exos (Exo-i-motif) to delivery DOX for anti-proliferation activity. The results indicated that Exo-i-motif showed significant anti-proliferation effect in MCF-7/MDR cells. Meanwhile, hypoxia is another feature of ITME and it can promote the tumor growth (149). In this situation, the reactive oxygen (ROS) secreted could correct hypoxia in TME and suppress cancer cells. Therefore, targeting acidic TME and correcting the hypoxic TME is also a promising approach for cancer therapy. Based on these, Zhang et al. (136) established a novel pH/light sensitive drug delivery platform using milk-Exos (Exo@DOX–EPT1) in squamous cell carcinoma therapy, and the scheme is shown in **Figure 4C**). In their work, DOX was conjugated to the membrane of Exos by a pH-cleavable bond which can target acidic microenvironment. Endoperoxides and Ce6 were both incorporated inside the Exos. The results indicated that Exo@DOX–EPT1 can be efficiently accumulated in tumor site and DOX was specifically released by acid environment stimulation. Ce6 could produce plasmonic heat upon NIR irradiating and ROS was effectively released to kill cancer cells.

#### 3.3.2 Immune checkpoint blockade

**Figure 5:**
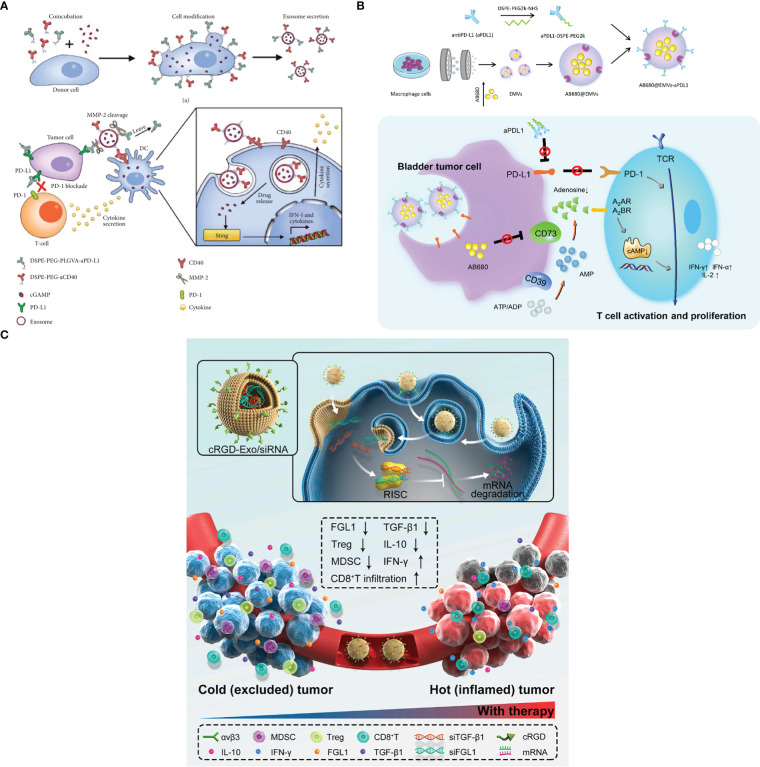
**(A)** The fabricated scheme of cGAMP@dual-anti-Exos and the process of cancer immunotherapy (Reprinted with the permission from Ref. 137). **(B)** The scheme of AB680@EMVs-aPDL1 for bladder cancer therapy (Reprinted with the permission from Ref. 153). **(C)** The mechanism of cRGD-Exo/siMix for colorectal cancer immunotherapy *in vivo* (Reprinted with the permission from Ref. 154). PD-1; Programmed cell death protein 1, PD-L1; Programmed cell death ligand; MMP-2; Matrix metalloproteinase enzyme; cGAMP; 2’-3’-cyclic guanosine monophosphate–adenosine monophosphate; AB680; CD73 inhibitor, EMVs; Exosome-mimetic nanovesicles, aPDL1; Monoclonal antibody targeting programmed cell death ligand 1; cRGD-Exo/siMix; a cyclic RGD peptide (cRGD)-modified exosome delivery system that simultaneously delivered FGL1 and TGFβ1 siRNAs.

ICB as an emerging cancer immunotherapy can block the regulatory receptors which are expressed on immune cells or tumor cells, and thus activating antitumor cytotoxic T-cell responses and improving cancer therapy efficiency (110, 150). In the past years, PD-1 and CTLA-4 inhibitory receptors were extensively studied and undergone clinic success (151). Despite ICB showed excellent cancer therapy effects, and some inhibitors including anti-CTLA-4 and PD-L1 monoclonal antibodies have been approved by FDA, however, some limitations still exist such as high off-target, low objective response rate and the risk of immune-related side effects (152). Therefore, in order to solve aforementioned drawbacks, many researchers focus on ICB inhibitors DDSs. For instance, Fan et al. (137) developed an Exos-based DDSs (named as cGAMP@dual-anti-Exos) in which anti-PD-L1 and anti-CD40 were all engineered on the surface of Exos for cancer immunotherapy, and the scheme is shown in **Figure 5A**). Firstly, lipophilic DSPE-PEG-anti-CD40 and DSPE-PEG-PLGVA-anti-PD-L1 were synthesized and applied to donor cells. Meanwhile, immune drug (2’-3’-cyclic guanosine monophosphate-adenosine monophosphate (cGAMP)) was also incubated with donor cells. And then, cGAMP@dual-anti-Exos was generated with these molecules loaded inside. The results indicated that cGAMP@dual-anti-Exos presented excellent targeting and anti-tumor effects, since PLGVA peptides could be cut off by the matrix metalloproteinase enzyme (MMP-2) inside the TME, and anti-PD-L1 was separated from Exos to achieve ICB (137). Similarly, Zhou et al. (153) designed and fabricated exosome-mimetic nanovesicles co-loading CD73 inhibitor (AB680) and PD-L1 antibodies (AB680@EMVs-aPDL1) to target bladder cancer, and the scheme is presented in **Figure 5B**). In this work, macrophage cell line (RAW264.7 cells) was chosen to secret exosome-mimetic nanovesicles and AB680 was loaded inside by coextrusion method. After that, PD-L1 antibodies was conjugated to the surface of the exosome-mimetic nanovesicles for ICB. The results suggested that AB680@EMVs-aPDL1 was conducive to drive the transition of CD8^+^ T-cells into effector cells owing to the existence of CD73 molecules. Moreover, a more efficient antitumor effect to PD-1 inhibition and better tumor regression were presented owing to a higher CD8^+^/CD4^+^ ratio in bladder cancer. In addition, the toxicity and biosafety *in vivo* were also evaluated, indicating that AB680@EMVs-aPDL1 was safe and had low toxicity. This work also provides a new and useful strategy for bladder cancer immunotherapy.

CTLA-4, which belongs to the CD28 receptor family, is overexpressed on the activated T cells and Tregs (10). It interacts with CD80/CD86 molecules expressed on the APCs and impedes T-cell activation and downregulates immune responses (21). Therefore, blocking the interaction between CTLA-4 and CD80/CD86 molecules on the APCs can activate T cells and enhance tumor immunotherapeutic efficacy. Recently, many researchers focus on this therapeutic strategy. For example, Phung et al. (10) constructed an exosome-based drug delivery platform (EXO-OVA-mAb) in which Exos were secreted from DCs and anti-CTLA-4 antibody was modified on their surface. EXO-OVA-mAb presented stronger ability of activating T cells than others and increased the CTLs/Treg ratio within the tumor site, and this phenomenon may be attributed to the crucial role of anti-CTLA-4 antibody. Moreover, EXO-OVA-mAb also increased the level of IFN-γ and TNF-α in both serum and tumors, and thus enhancing cancer therapeutic effect.

CD47 as another immune checkpoint is also overexpressed on the most tumor cells, and it often interacts with signal regulatory protein α (SIRPα) on phagocytic cells, which activates “don’t eat me signal” of CD47 and leads tumor cells to escape from immune monitoring (16). Therefore, blocking the interaction between CD47 and SIRPα can enhance tumor therapeutic efficacy. Based on this strategy, Koh et al. (155) designed and developed SIRPα-Exos for interfering CD47-SIRPa interaction to enhance cancer immunotherapy. In their work, plasmid DNA encoding SIRPa variant was firstly constructed and cocultured with HEK293T cells. And then, engineered Exos with SIRPα proteins were obtained by ultracentrifuged method. Finally, the anti-tumor effect was evaluated in mouse model. The results indicated that SIRPα-Exos presented higher CD47 affinity than control Exos, and enhanced tumor cell phagocytosis *in vitro* and *in vivo*. In addition, the existence of SIRPα-Exos also improved the infiltration of CD8^+^ T cell, suggesting that SIRPα-Exos could efficiently induce tumor phagocytosis and lead to anti-tumor T cell response.

In addition, silencing the expression of tumor immune checkpoint is also another strategy for cancer immunotherapy. Pei et al. (154) established a cyclic RGD peptide (cRGD)-modified exosome co-loaded with siFGL1 and siTGF-β1 (cRGD-Exo/siMix) for colorectal cancer immunotherapy by ICB, and the scheme is shown in **Figure 5C**). cRGD-Exo/siMix can efficiently deliver siFGL1 to silence the expression of tumor immune checkpoint ligand FGL1, and T cells were significantly activated.

#### 3.3.3 Exosomes-based therapeutic cancer vaccines

It is well known that cancer immunotherapy is largely dependent on the functions of APCs and T cell, because the cancer immunity cycle must be repeatedly initiated and expanded to achieve sustained cancer immune response. In view of this, many researchers focus on cancer immunotherapy *via* Exos-based therapeutic cancer vaccines.

**Figure 6:**
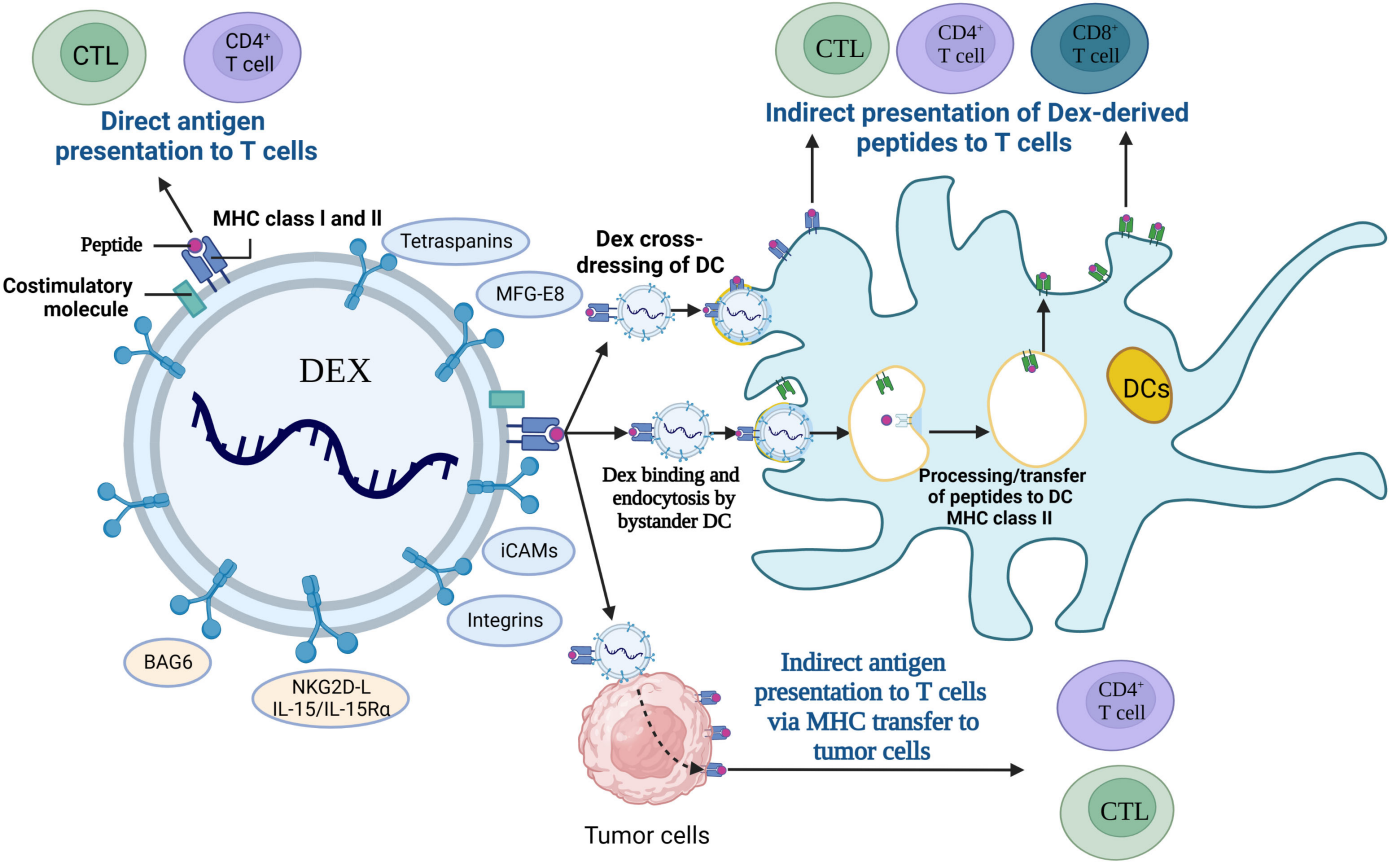
Interaction of DEXs with immune cells. CTL; cytotoxic T lymphocyte, DCs; Dendritic cells, MFG-E8; milk fat globule EGF factor 8, iCAMs; intercellular cell adhesion molecules, BAG6; Bcl-2-associated athanogen-6, NKG2D-L; natural killer group 2, member D receptor ligands, Dex; DC-derived exosomes, MHC; major histocompatibility complex.

DEXs have been widely used in therapeutic vaccines as an effective alternative to tumor antigens and have tremendous potential for cancer immunotherapy due to their features of long validity period and easily being engineered (156). DEXs express peptide/MHC-I and peptide/MHC-II complexes (pMHC I and pMHC II), heat-shock proteins (HSP), costimulatory molecules (CD80, CD86) and adhesion molecules, and they are involved in antigen uptake and presentation, and also activation of the antitumor response in CD4^+^ and CD8^+^ T cells (157), and the interaction mechanism is shown in **Figure 6**. Research found that Exos secreted from α-fetoprotein (AFP)-expressing DCs (DEX_AFP_) stimulated CD8^+^ T lymphocytes to express IFN-γ and secrete IL-2, which leaded to the reduced CD25^+^Foxp3^+^ Treg, IL-10 and TGF-β in the tumor microenvironment (158). DEX_AFP_ elicited potent antigen-specific immune responses and was proved to be a cell-free vaccine for immunotherapy. Furthermore, a novel EXO-T vaccine was developed which converted the exhausted T cells into tumor-specific effector CTL *via* the CD40L signaling pathway of CD4^+^ T cells to stimulate a more massive CTL anti-tumor response (159). Moreover, HER2-specific exosome (EXO)-T vaccine was also developed to trigger the activation of immune responses and assist in the treatment against HER2-positive breast cancer (160).

**Figure 7:**
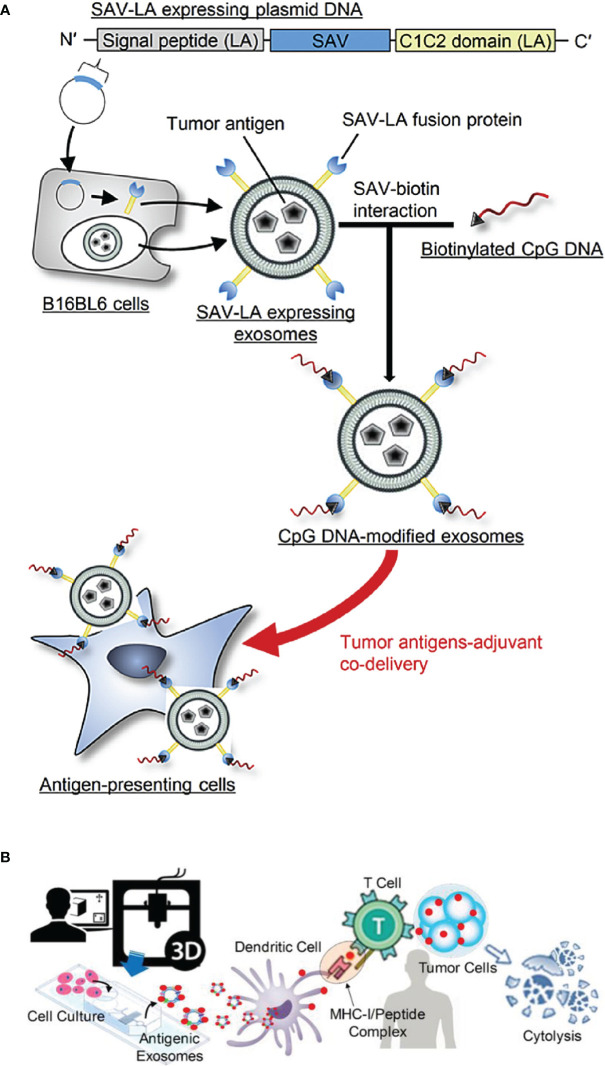
**(A)** The scheme of CpG-SAV-exo to deliver APCs (Reprinted with the permission from Ref. 9); **(B)** The scheme of MHC-I positive exosomes for activating anti-tumor responses (Reprinted with the permission from Ref. 171). SAV; Streptavidin, SAV-LA; N-terminal secretion signal of lactadherin (LA) and C1C2 domain of LA.

In general, TEXs can also interfere with the immune system by delivering tumor antigens to DCs. However, because TEXs have the dual role of immunosuppressive and immune activating effects, there is a concern that TEXs will block antigen processing and presentation in DCs (161–164). Study showed that TEXs could be used for vaccine with immunostimulatory effects because they have the same rejection antigens as tumor cells (165). Recently, TEXs containing tumor-specific antigens were extracted from autologous tumor tissue to regulate the Th1 immune response in melanoma, and they blocked tumor growth and metastasis (166). However, the immune response elicited by TEXs is relatively weak which results in the unsatisfactory antitumor effect, so efforts have been made to generate vaccine systems, such as artificially modified TEXs and TEXs-loaded DC, with higher immunogenicity, (167). Common strategies for TEXs modifications include genetic modification (168), external stimulation of donor cells (169), and incorporation of fusion proteins (170). CIITA (Class II transactivator) gene was transduced into B16F1 murine melanoma cell line (B16F1- CIITA) by genetic engineering, and the secreted Exos (CIITA- Exo) expressed high level of MHC-II as well as the tumor antigen TRP2. CIITA-Exo enhanced the splenocyte proliferation and IL-2 secretion, and induced inflammatory cytokines (such as TNF-α and IL-12) mRNA production, so that CIITA-Exo had a more potent anti-tumor immune response compared to control Exos (168). In addition, Morishita et al. (9) chose TEXs as tumor antigen carrier to establish a tumor antigens-adjuvant co-delivery system. In their work, firstly, murine melanoma B16BL6 tumor cells were engineered to produce Exos expressing SAV-LA, and then immunostimulatory CpG DNA was modified on the surface of Exos by SAV-biotin interaction (CpG-SAV-Exo), and the scheme is shown in **Figure 7A**). The results indicated that CpG-SAV-Exo could efficiently deliver CpG DNA to APC, showing a high antigens-presenting capacity. Meanwhile, CpG-SAV-Exo can efficiently activate T cells and present an excellent antitumor efficacy. Apart from genetic modification, to enrich Exos with more HSP70, external heat stimulation was applied to tumor cells, and the HSP70-enriched Exos (HS Exo) was shown to increase the expression of MHC-II and achieve higher productions of IgG2a and IFN-γ, resulting in strong Th1 immune responses and eliminating cancer cells (169). In addition, the incorporation of viral fusion proteins (such as the G protein of vesicular stomatitis virus (VSV-G)) into TEXs enhances their uptake, induces the maturation of DCs, and improves immunogenicity (172). Co-expression of antigen OVA and VSV-G on TEXs induced a specific CTL immune response *in vivo*, as exhibited with increased IgG2a antibody responses and amplification of antigen-specific CD8^+^ T cells (170). In addition, another strategy to enhance TEXs vaccine activity is the application of TEXs-loaded DCs, which is due to the advantage of efficient antigen processing and MHC I loading of DCs after co-incubation with TAA-TEXs. Therefore, *in vitro* activation and loading of TEXs into DCs initiate an effective antitumor response, which overcomes the immunosuppressive limitations of TEXs alone (165). TEXs-loaded DCs activate T lymphocytes to develop into antigen-specific CTLs and trigger specific CTL immune responses with strongly cytotoxicity to autologous tumor cells (173). In a similar study, DCs that loaded with Exos from the supernatant of HeLa cells (HeLa-TEXs) enhanced the proliferation and cytotoxic activity of CTLs, whereas HeLa-TEXs alone showed no effect (174). Before TEXs-loaded DCs were developed, DCs were also used to load tumor lysates, but there is no disputing that TEXs are a better source of TAA due to the better antigen processing and presentation (175). In a comparative study, TEXs-loaded DCs (DC-TEXs) was significantly superior to lysate-loaded DCs in vaccination efficacy. TEX is more effective than tumor lysates in inducing an appropriate anti-tumor immune response, avoiding potentially fatalities in inoculated mice, and providing more persistent antigen presentation and priority antigen processing (176). Overall, immunogenic Exos could serve as adjuvants for therapeutic cancer vaccines in the future.

In addition, a novel strategy for directly activating T cells was also introduced in recently years. Zhao et al. (171) designed and developed a microfluidic device to produce antigenic Exos modified with peptide complex (e.g., gp-100, MART-1, and MAGE-A3) on demand. They also designed magnetic-nanoparticles with functionalized photo-cleavable and peptide affinity probe for capturing antigenic Exos *via* a light trigger. Meanwhile, the antitumor capability of antigenic Exos was also evaluated *in vitro* and *in vivo*, and the scheme is shown in **Figure 7B**). The results indicated that Exos which were modified with melanoma tumor peptides including gp-100, MART-1 and MAGE-A3 enhanced the ability of antigen presentation and T cell activation. This is because MHC-I and tumor peptides can form MHC-I/peptide binding complex which can be presented to cytotoxic T cells and thus triggering an immediate response from the immune system (3, 171). Moreover, conjugating cytokine-loaded Exos to T cells surfaces is also another strategy that can enhance adaptive T cell therapy. This approach is simple and can minimize systemic side effects of adjuvant drugs (21).

#### 3.3.4 Combination therapy

**Figure 8:**
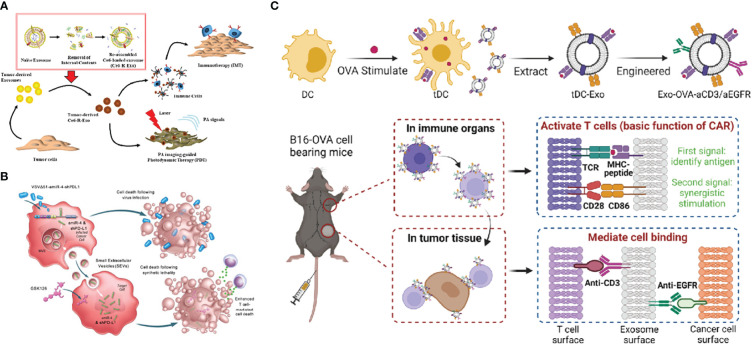
**(A)** Schematic diagram of photoacoustic imaging-guided combined photodynamic and immunotherapy for Ce6-R-Exo treatment (Reprinted with the permission from Ref. 191) **(B)** Schematic illustration of VSVΔ51-amiR-4-shPD-L1 exerting enhanced T cell-mediated cancer cell death (Reprinted with the permission from Ref. 192) **(C)** The above diagram is a schematic view of the construction of the engineered tDC-Exo (Exo-OVA-aCD3/aEGFR) with anti-CD3 and anti-EGFR antibodies. The bottom diagram shows the simulated CAR-T treatment process (Reprinted with the permission from Ref. 193). R-Exo; re-assembled exosome, Ce6-R-Exo; chlorin e6-loaded R-Exo, IMT; immunotherapy, PDT; photodynamic therapy, PA; photoacoustic, VSVΔ51-amiR-4-shPD-L1; VSVΔ51 oncolytic viruses- artificial microRNA-4- shPD-L1, MVB; multivesicular bodies, SEVs; small extracellular vesicles, DC; dendritic cells, tDC-Exo;tumor antigen-stimulated dendritic cell-derived exosomes, aEGFR; anti-epidermal growth factor receptors antibodies, OVA; ovalbumin, CAR; chimeric antigen receptor, TCR; T cell receptor, MHC; major histocompatibility complex.

The development of immunotherapy has yielded remarkable results in recent years. Currently, various ICIs have been approved by FDA as single agents for cancer treatment, however, the response rate for ICIs is only 10-35% (177–179). The effectiveness of immunotherapy is directly dependent on the state of the tumor microenvironment, while TME mostly presents an immunosuppressed condition with lack of T-cell infiltration or dysfunction, poor immunogenicity. Moreover, multiple mechanisms of drug resistance also contribute to the low efficiency in immunotherapy (180). Therefore, new alternative treatment strategies are being explored, and combination therapy containing two or three anti-tumor approaches (including chemotherapy, radiotherapy, photodynamic therapy, targeted therapy, vaccines, oncolytic viruses, ICB, ACT etc.) to achieve higher efficacy is under evaluation (181, 182). Chemotherapeutic agents [such as anthracyclines, cyclophosphamide, oxaliplatin and paclitaxel (183)] are highly cytotoxic. However, chemotherapeutic drugs can trigger immunogenic cell death to act as adjuvants for immunotherapy by releasing damage-associated molecular patterns and activating apoptosis which make tumors more sensitive to immunotherapy (184). TEX-loaded DC vaccine in combination with chemotherapy could effectively suppress tumor-infiltrating MDSCs, inhibite tumor cell migration and promote greater T-cell activation, resulting in a longer survival time compared to DCs-TEX vaccinated only mice (185). Likewise, radiotherapy can enhance the antitumor effects of immunotherapy by increasing tumor antigenicity through multiple approaches. Radiation has an abscopal effect allowing for systemic tumor control (186) and can trigger the cGAS/STING pathway and stimulate innate and adaptive immune responses through DNA damage and ROS production (187). Short-burst radiation treatment significantly enhanced the delivery efficiency of PD-L1siRNA-loaded targeted Exos, altered the immune environment, sensitized poorly immunogenic glioblastomas to ICB, inhibited tumor growth, and prolonged the survival of tumor-bearing mice (188, 189). Otherwise, photodynamic therapy in synergy with immunotherapy has become a focus of research to overcome the low efficacy of immunotherapy for primary tumors and to monitor the drug delivery status at the target site (190). TEXs loaded with the photosensitizer Ce6 have been used as vehicles for photoacoustic-guided photodynamic therapy and as tumor antigens to stimulate the immune system to activate anti-tumor responses (191), and the scheme is illustrated in **Figure 8A**. The lack of tumor infiltration in ICB can also be addressed by oncolytic viruses, which provide a critical switch for the immune system. Oncolytic viruses invade tumor cells and replicate extensively inside, leading to tumor cell lysis (194), and on the other hand recruiting TILs into the damaged tumor, initiating the release of tumor antigens and pro-inflammatory cytokines and promoting the activation of the immune system (195). The scheme is shown in **Figure 8B**, and it was demonstrated that VSVΔ51 oncolytic viruses loaded with artificial amiRNA-4, when co-targeted with Exos carrying amiRNA-4 and PD-L1 shRNA cargoes, upregulated PD-L1 expression, sensitized tumors to CTLA4 and PD-1 immune checkpoint inhibition, enhanced death of tumor cells, and prolonged overall survival in mice (192). Chimeric antigen receptor T (CAR T) cell therapy has achieved remarkable results in hematologic malignancies, but the results in solid tumors have been less than satisfactory. TDC-Exo, a DC-secreted exosome stimulated by tumor antigen carrying MHC-antigen complexes and CD86, was developed as the “CAR” portion of CAR-T, activating T cells and cooperating with anti-CD3 and anti-EGFR and immune checkpoint inhibitory antibodies anti-PD-L1, further enhancing the efficacy of the CAR-T cell therapy mimetic platform for solid tumor treatment (193). The scheme is demonstrated in **Figure 8C**.

## 4 Clinical translation and challenges of Exos as DDSs

Currently, several cancers therapeutic strategies-based Exos DDSs have undergone clinic trials, and the relevant data which was obtained from https://clinicaltrials.gov/ are present in **Table 3**. As shown in **Table 3**, only few of Exos-based DDSs for cancer therapy have entered into clinical trials. Moreover, all of them are in the early clinical stage, suggesting that they still face many challenges.

**Table 3:** The current clinical trials of Exos as drug delivery vehicles.

DDSs	Exos source	Disease	NTC number	Clinic phase
Curcumin Exos	Plant	Colon cancer	NCT01294072	Phase 1
A vaccination with tumor antigen-loaded Exos	Dendritic cell	Non-small cell lung cancer	NCT01159288	Phase 2
Exos with KRAS G12D siRNA (iExos)	Mesenchymal stromal cells	Pancreatic cancer	NCT03608631	Phase 1

The data is obtained from https://clinicaltrials.gov/.

Firstly, the stable mass production of Exos is the primary challenge. As is known to all, the selection and culture of donor cells are one of significantly important factors. In recent years, mesenchymal stromal cells and cardiac progenitor cells have been proved to provide stable Exos production during scale-up culture. Moreover, cell culture technologies have also been improved and up to 20,000 L of cells can also be cultured *via* stainless-steel bioreactors. In spite of this, the clinical translation of Exos is still difficult. The main reason is that the scaling-up process is relatively expensive. Furthermore, the conditions of cell culture also need to be meticulous, because improper operation may cause cell contamination, which can result in cell subtypes and variation. Therefore, strictly controlling and maintaining the genetic stability of donor cells are difficult.

Secondly, the isolation and purification of Exos are another challenge. Currently, the extraction technologies including ultracentrifugation, tangential flow fractionation, exclusion chromatography and commercial extraction kits have been extensively employed to isolate Exos. Of which, tangential flow fractionation is often used in the mass production of Exos in the clinical trials. However, the purity of Exos obtained by this separation method is low, thereby limiting its application. Although high purity of Exos can be obtained by ultracentrifugation, its features of low throughput and high cost limit the mass production of Exos. Currently, there is no standard procedures for large-scale Exos separation. Therefore, it is urgent to develop an advanced technique with high efficiency, high quality and low cost to separate Exos for DDSs.

Furthermore, the surface modification of Exos is also one of important factors because it affects the targeting functions and biological effects of DDSs. In general, two methods including chemical modification and genetic engineering can be used for the surface modification of Exos. Genetic engineering is highly effective for surface modification by fusing the gene sequence of targeting protein with exosomal membrane protein. However, this approach is limited to genetically encoded targeting motifs. Chemical modification often affects the structure and function of Exos, and thus limiting their application. Meanwhile, there is no standard strategies for loading drugs. Recently, many drug-loading strategies including incubation, electroporation, ultrasound, and cell engineering techniques have been applied. They all have their limitations to some extent. For instance, incubation is the simplest drug-loading method. It does not require special equipment, and the structure of Exos is rarely damaged. However, low drug-loading efficiency was presented in this loading method. Although ultrasound and electroporation can improve drug-loading efficiency, the membrane of exosome maybe damaged and aggregation of Exos may be caused by these methods. Meanwhile, the operation of cell engineering techniques is too complicated though it is considered as well-established operating strategy.

In addition to these limitations mentioned above, the storage conditions of purified Exos also play critical role in clinical translation of Exos. An increasing number of researches suggested that Exos derived from different sources require different storage conditions, because the storage temperature and storage solution (e.g., saline, PBS, cell culture media, etc.) all affected the particle size and protein content of Exos. Therefore, further researches should take the influences of storge conditions into consideration for Exos as drug delivery carriers.

## 5 Conclusions

In this review, some relevant knowledges including the biogenesis and composition of Exos, the source of Exos for DDSs, drug-loading strategies, cancer immunotherapy strategies, and their clinical translation and challenges were discussed. Exos are mainly divided into immune cell-derived and tumor cell-derived Exos. They can inherit the properties of donor cells and participate in the innate and adaptive immune responses, thus promoting extensive applications of immune cells-derived Exos as drug delivery carrier. Meanwhile, various drug-loading strategies of Exos-based DDSs including incubation, physical loading techniques (e.g., electroporation, ultrasound and extrusion), and cell engineering techniques have been designed and developed. In addition, Exos-based cancer immunotherapy strategies (e.g., tuning of ITME, ICB, cancer vaccines, etc.) have been extensively applied, they all presented excellent therapeutic effects.

To our delight, nowadays, several cancers immunotherapeutic strategies-based Exos DDSs have undergone clinic trials. In spite of this, they still face many challenges including their stable mass production, their isolation and purification, their surface modification, and their storage conditions. Therefore, cell culture technologies should be further improved and related bioreactors should also be designed and developed to scale up the Exos production in the future. In addition, it is urgent to develop an advanced technique with high efficiency, high quality and low cost to separate and purify Exos which can be used in DDSs. Meanwhile, the storage conditions of purified Exos from different cell sources should be further explored. Overall, although Exos as drug delivery vesicles still exist some challenges, they provide an excellent platform for cancer immunotherapy.

## Author contributions

All authors listed have made a substantial, direct, and intellectual contribution to the work and approved it for publication.
